# Analysis of Machine Learning–Based Investigation Into Multivariate Factors of Team Performance in Serious Games: Cross-Sectional Retrospective Study

**DOI:** 10.2196/83478

**Published:** 2026-04-13

**Authors:** Gruyff Germain Abdul-Rahman, Freark de Lange, Andrej Zwitter, Noman Haleem

**Affiliations:** 1 Department of Responsible Governance and Technology, Campus Fryslân, University of Groningen, Wirdumerdijk 34, Leeuwarden, 8911 CE, The Netherlands, 44 7533786621; 2 Centre for Human and Data Sciences, University of Klagenfurt, Lakeside Park B07b.01.121Klagenfurt, Carinthia, 9020, Austria

**Keywords:** serious games, team performance, escape rooms, machine learning, explainable AI, explainable artificial intelligence

## Abstract

**Background:**

Serious games (SGs) are increasingly used to study and enhance team performance in organizational and educational settings. While prior research has explored leadership and communication as isolated factors, the multivariate interactions between behavioral indicators remain poorly understood. A deeper understanding of these relationships can reveal which behavioral and demographic factors most strongly predict successful outcomes, offering insights relevant to both scientific research and practical training design.

**Objective:**

This study aimed to develop machine learning (ML) models to predict team success in SGs. Specifically, it sought to identify the behavioral and demographic predictors that most strongly influence team performance outcomes.

**Methods:**

This study used a cross-sectional retrospective design. Behavioral and demographic data were analyzed from 233 teams participating in escape room–based SGs delivered by JGM Serious eXperiences in The Netherlands. Teams of 2‐8 players (mean age 25.8 y; 53 all-male, 55 all-female, and 125 mixed-gender) were scored by trained observers across collaboration, communication, and leadership constructs using Likert-scale indicators. Exploratory data analysis compared winning (n=141) and losing teams (n=92) using descriptive statistics, Pearson correlations, and significance testing (independent-samples *t* tests and Mann-Whitney *U* tests). Mean differences were interpreted with 95% CIs. A total of 4 ML models: logistic regression, random forest, multilayer perceptron, and support vector classifier, were trained using 5-fold cross-validation (*F*_1_-score). The best model was interpreted using SHAP (Shapley Additive Explanations).

**Results:**

Winning teams scored higher on several behavioral constructs, but only 4: knowledge sharing, leadership, guidance, and extraversion, showed statistically significant differences between winners and losing teams. These effects were supported by 95% CIs, Shapiro-Wilk tests for normality, and Mann-Whitney *U* tests where assumptions were violated, indicating that only a subset of behavioral indicators meaningfully distinguishes successful teams. Among the ML models, logistic regression achieved the highest accuracy (88%), followed by multilayer perceptron (87%), random forest (87%), and support vector classifier (85%). SHAP analysis showed that gender composition and prior escape-room experience were the strongest demographic predictors of success, while “celebrating progress” (extern5) and “taking initiative when the team is stuck” (sturing5) were the most influential behavioral indicators.

**Conclusions:**

This work demonstrates the usefulness of multivariate analysis in studying and understanding complex human behavior in SG environments as opposed to studying isolated behavioral indicators, often described in previous studies. The ML models developed using behavioral and demographic features of participating teams showed promising accuracies, and their interpretation led to unveiling a set of demographic and behavioral components as the most decisive factors leading to team success. This improved understanding of what makes a team win can be potentially translated into terms of improved productivity in business and organizational settings.

## Introduction

Recent advances in data analytics have facilitated machine learning (ML) applications across several domains of science and business. A total of 1 area that has also revolutionized significantly from these developments is sports and gaming [1], where ML techniques can reveal hidden trends [2] that are important for the development of modern sports and provide a glimpse into the intricacy of player performance [1]. Predicting outcomes based on historical evidence has long been a central goal for researchers. This motivation has driven the development of various approaches, including sport-specific statistical simulations and sport-independent machine-learning techniques [2]. By using these tools, trends in sports data can be identified and used for personal, competitive, or economic advantage [2]. By using cutting-edge algorithms, sports analysts can uncover insights that were previously unattainable, ranging from physical attributes to strategic patterns, ultimately enabling a more comprehensive understanding that surpasses conventional analytical techniques [1]. The early work in this regard dates back more than half a century, when Samuel [3] used ML for game strategy optimization, marking the intersection of artificial intelligence (AI) and gaming. More recently, ML models have been applied to predict individual and team performance using historical data, which has enabled refined decision-making in competition [4,5]. For example, in football, variables such as shots on target, ball possession, and passing strategies were studied using ML techniques and subsequently linked to team success [6]. In cricket and basketball, ML models, such as Naive Bayes (NB) and support vector machines (SVMs), have demonstrated high predictive accuracy by accounting for complex interactions between team composition and match conditions [7,8]. ML techniques have also been effectively applied to understand team performance in sports, with methods such as random forest (RF) and Partial Least Squares Discriminant Analysis being applied to identify key performance metrics [9]. On an aggregate level, the application of ML has shown a promising potential in game analysis by capturing intricate performance patterns, which were otherwise difficult to analyze using traditional statistical techniques.

Recent work in the field of serious games (SGs) reflects a similar shift toward data-driven analysis and adaptive systems. Although most SGs do not currently incorporate ML, there is a growing trend toward integrating ML to assess participants and automatically adapt gameplay to individual needs [10]. These adaptive systems can personalize difficulty, improve engagement, and reduce learner frustration, but they also introduce practical challenges, such as selecting appropriate data sources, determining which game elements to modify, resolving cold-start problems, and validating whether adaptive changes genuinely support learning [10]. Evidence shows that ML techniques are increasingly used in SGs to monitor learner behavior, generate real-time feedback, and model player states such as motivation, skill level, or emotional reactions [11,12]. This has led to the emergence of AI-powered SGs that adjust game content based on individual progress, preferences, and training goals, thereby creating more personalized and effective learning experiences [13,14].

A recent systematic review highlights that advanced technologies, such as AI, immersive environments, and biosignal monitoring, can enhance meta-skills training by supporting adaptive learning, targeted practice, and improved monitoring of behavioral and cognitive states [15]. Studies demonstrate that AI-based SGs can develop key meta-skills, such as time management, self-regulation, and motivation, through difficulty adjustment and personalized support. For example, a study [16] used ML models to analyze players’ biosignals in real time and modify game difficulty based on their emotional state, resulting in improved time-management skills. Despite these benefits, the integration of AI into SGs also raises several concerns. The development of AI-powered games requires significant technological infrastructure and expertise [11]. Concerns have been raised regarding data privacy, potential algorithmic biases, and inequities related to access and fairness [15,17]. To address these challenges, recent literature emphasizes the need for ethical design principles, secure data practices, and transparent model development to ensure trustworthy and inclusive applications of AI in SGs.

AI-based SGs offer various potential benefits for meta-skills training, but they also introduce important challenges that require mitigation. One key advantage is improved access to personalized training, as AI can adapt learning content and game difficulty to individual player capabilities and provide instant feedback with continuous performance tracking. AI systems can also support a deeper understanding of teamwork, communication, leadership, and behavioral patterns and enable training to be delivered at scale, at low cost, and remotely. However, concerns remain about how AI systems make decisions, the risk of confusing or misleading players, and an increasing dependence on automated feedback. Additional challenges include reduced human interaction, risks related to storing and handling sensitive data, and the possibility of biased or unfair outcomes if AI models are trained on unrepresentative datasets. To address these issues, the literature highlights the importance of transparent and responsible AI design, the use of simple and explainable adaptation rules, balancing automated feedback with human guidance, and maintaining opportunities for reflection and social interaction. Robust data protection measures, including encryption and restricted access, as well as regular bias checks using diverse datasets, are also essential to ensure ethical and effective deployment of AI-based SGs [11,14,15,17-21].

SGs are interactive experiences created with objectives that extend beyond mere entertainment, serving functions such as education, skill development, or problem-solving [22]. They typically represent real-world systems or processes in a simplified manner, enabling participants to engage with and better understand the complexities of real-life structures through a distilled, manageable format [23]. One common implementation of SGs is through escape rooms, where groups of individuals collaborate to solve complex puzzles under given time constraints. During such SGs, an expert technician monitors the teams and assesses their behavioral and psychological attributes and constructs, for example, the abilities of collaboration, decision-making, and leadership. These attributes are often quantitative as scores are assigned on a Likert scale. The primary game outcome, that is, if the team completed the task within the allotted time, is also recorded. This data is then analyzed through a variety of methods to explore how different team attributes and compositions influence their performance and game outcome. With a promising potential to provide insights into the factors that contribute to team success, SGs are now increasingly recognized as a tool for enhancing teamwork, collaboration, and problem-solving [24].

Despite significant progress in general sports analytics [7,25], the application of ML in analyzing team performance within serious gaming environments and escape rooms is rather limited. The data acquired through escape rooms is usually analyzed using traditional statistical methods, for example, 1-way ANOVA, which cannot capture the multivariate nature of several variables and their interactions, limiting our ability to achieve deeper and accurate insights into complex team behaviors [9]. Some previous studies on serious gaming have focused on aspects such as team cohesion and leadership dynamics [26,27], but the quantification of specific behaviors contributing to team success is not yet fully understood. The growing body of literature on ML in sports analytics already highlights the utility of multivariate models in understanding team performance [4], but its application in serious gaming is yet to be realized. This gap highlights the need to apply data-driven, multivariate analytical approaches to understand the factors that drive team success. This knowledge will eventually contribute to forming better teams, which could lead to improved efficiency and productivity in organizational settings.

In this study, we aim to address the above-described research gap by applying exploratory and ML techniques to identify the multivariate feature combinations which influence team performance in serious gaming, specifically within escape room settings. Various ML-based models were developed using a serious gaming dataset to study key indicators in team behavior and dynamics that can distinguish successful teams from unsuccessful ones. In addition, explainable AI methods were used to tease out the most significant feature combinations that played a decisive role in determining game outcomes. This knowledge can likely be translated into terms of understanding to develop more efficient and productive teams in organizational and business settings.

## Methods

### Data Collection

This study used a cross-sectional retrospective design, as the outcome data had already been collected, and the analysis focused on examining existing data to explore potential relationships. This study was conducted in collaboration with JGM Serious eXperiences, a provider of escape rooms and serious gaming environments designed for corporate and educational training based in Leeuwarden, The Netherlands. The research partnership began in November 2022 and included regular in-person meetings between the research team and JGM staff through July 2023. The concept of SGs, as implemented by JGM, involves using traditional entertainment games for educational and instructional objectives [24]. Using SGs for educational purposes makes it possible to measure how game-based activities influence participant behavior, supporting goals in education and organizational development.

The data collection process was carried out in multiple phases. First, an expert technician documents key demographic information before participants engage in the escape room experience. This includes variables such as age, gender distribution, team size, and prior escape room experience. These demographic factors provide context for the interpretation of behavioral patterns. Any personal data from the consent form of participants is stored in compliance with the relevant privacy regulations. Additionally, participation in JGM’s escape room is fully voluntary. Participants are asked to read and sign an informed consent form, and a verbal agreement is also explained to all participants during the introduction session. Participants are also free to withdraw at any time and are properly debriefed afterward.

Second, during the escape room activity, the actions of the participants were continuously recorded using closed-circuit television cameras. Alongside, the expert technicians rated predefined behavioral indicators on a Likert scale, focusing on three constructs: (1) collaboration, (2) communication, and (3) leadership. The behavioral model guiding these observations was adapted from the Anesthetists’ Non-Technical Skills (ANTS) Framework [28] and tailored for escape room settings in cooperation with psychologists and academics specialized in personal leadership. Although the ANTS Framework was designed for clinical settings, it draws on general teamwork and organizational behavior theory by focusing on observable behaviors that contribute to effective team performance [28]. The framework entails both interpersonal skills, for example, communication, teamwork, leadership, and cognitive skills, such as situation awareness and decision-making [28], which are critical in high-pressure collaborative environments such as escape rooms. In order to train nontechnical skills effectively, it is essential to first identify which skills are required in a specific environment and then assess them in a structured way. This makes it possible to give clear feedback and evaluate whether training leads to measurable improvement [28]. In collaboration with psychologists and experts in personal leadership, JGM adapted this framework to the escape-room environment, ensuring that each behavioral indicator reflected established constructs in teamwork research while remaining observable. This theoretical foundation helps connect our results to well-established models (such as the ANTS framework) of how teams operate.

Third, upon completion of the escape room session, the participants engaged in a structured reflection process. They reviewed the video recordings of their performance, received feedback from team members, and discussed insights from the analyzer report on aspects related to collaboration, communication, and leadership. The expert technicians also provided personalized feedback, highlighting strengths and areas for improvement on an individual basis. The disagreement of participants on a certain item in the feedback served as a catalyst for discussion on discrepancies between perceived and observed behaviors. Conversely, when there was an agreement, the focus shifted toward the strategies for translating these behavioral insights into real-world contexts, such as professional settings. This reflective exercise not only served as a learning intervention but also reinforced the reliability of the observations recorded by expert technicians.

**Figure 1.**
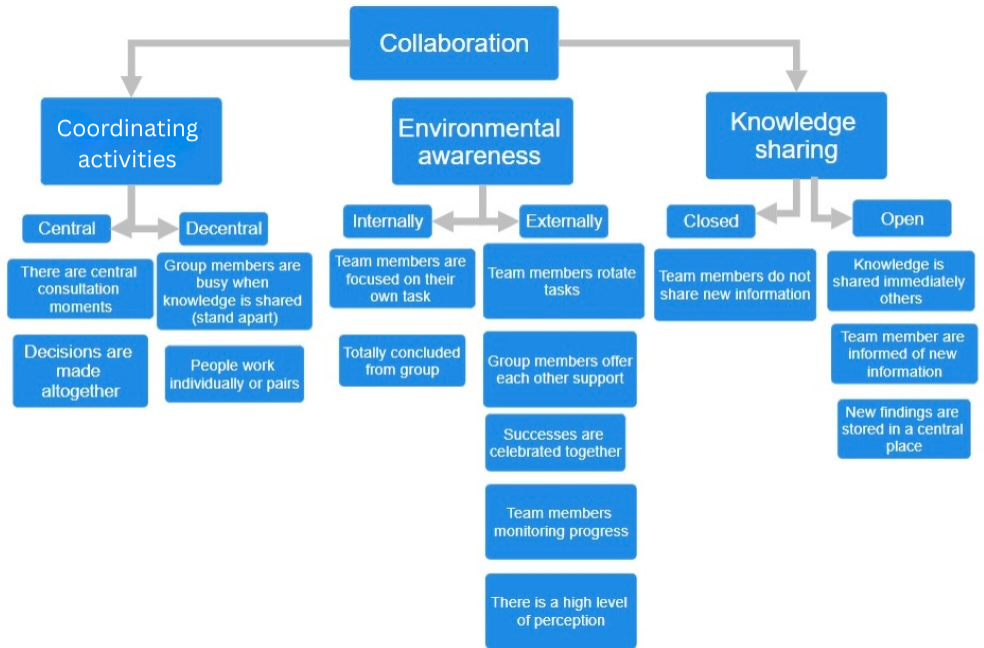
Graphical representation of the collaboration construct.

**Figure 2.**
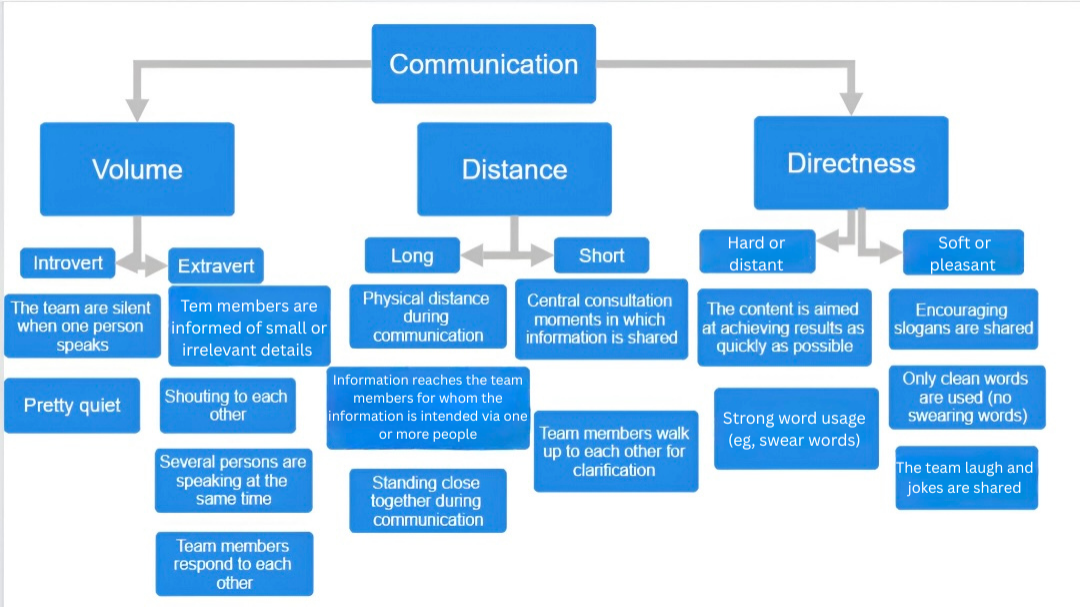
Graphical representation of the communication construct.

**Figure 3.**
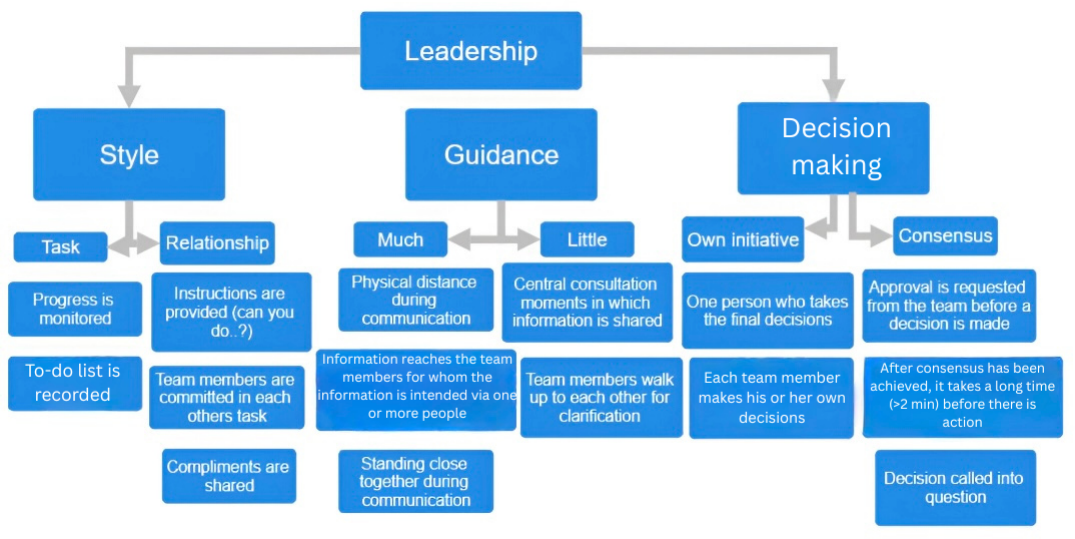
Graphical representation of the leadership construct.

Various behavioral components for which the data were recorded on a Likert scale are shown in Figures1^3^. Figure 1 depicts the visual representation of the collaboration construct, Figure 2 shows the communication construct, and Figure 3 shows the leadership construct. For example, Figure 1 illustrates the construct of collaboration, which includes behavioral components such as coordinating activities, knowledge sharing, and environmental awareness. Each of these components is assessed using 2 opposite indicators (central vs decentralized). These indicators are measured through specific variables, and the responses are used to calculate a score for each indicator. The scores reflect how well a team performs in each behavioral area. For instance, a score above 3.5 suggests strong team unity and coordination (central), while a score below 2.5 indicates a more individual, less coordinated approach (decentralized).

### Data Preparation and Exploratory Analysis

**Figure 4.**
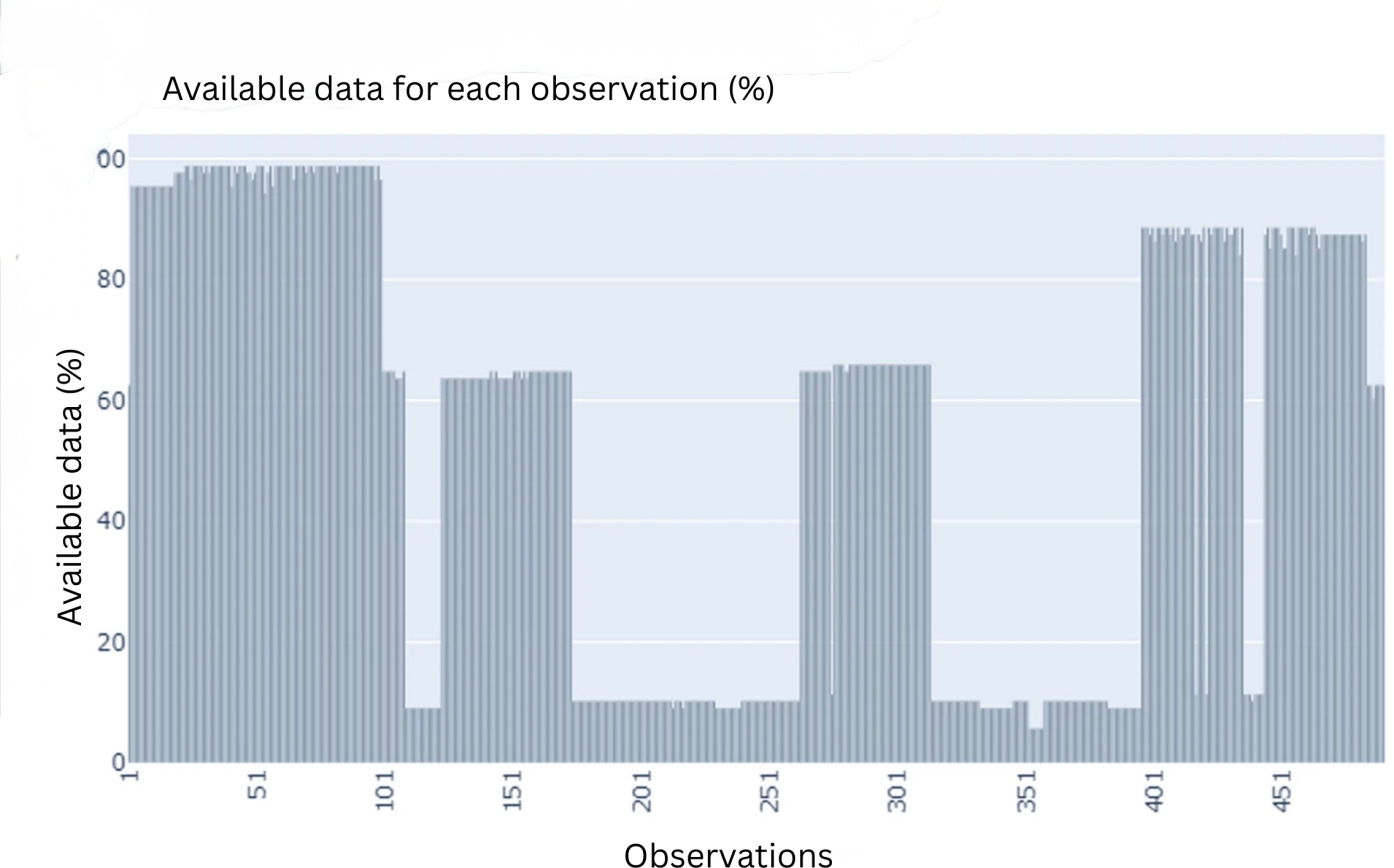
Visualization of the percentage of available data for each observation.

Once the collection of data had been completed, JGM’s expert technicians stored the dataset in Excel (Microsoft Corp) spreadsheet in compliance with privacy regulations. This resulted in a raw dataset containing 88 features, 490 rows, and 20,246 missing values. Figure 4 illustrates the percentage of available data per observation, grouped in bins of 50 observations. It indicates that missingness is not uniformly distributed across the dataset. The first 100 observations show very high data completeness, with approximately 95%‐100% available data. Several subsequent observation blocks (eg, around observations 100‐250 and 300‐380) show substantially lower completeness, often around 5%‐15% available data, indicating a high level of missingness in these cases. Other observation ranges (eg, around observations 250‐300 and 400‐470) show moderate to high completeness, typically between 60%‐90% available data. Overall, Figure 4 shows that missing values are unevenly distributed, with some observations containing substantially more missing data than others.

The data preparation was performed using the Pandas library (version 2.2.3) in Python (Python Software Foundation) programming language (version 3.13.1) in Jupyter notebooks. All analyses were performed on an AMD Ryzen (9 processor, 40 GB memory; Advanced Micro Devices, Inc) laptop running Microsoft Windows 11 Pro (version 24H2; Microsoft Corp), provided by University of Groningen (Campus Fryslân). All ML models were developed and tested using the SciKit-Learn (version 1.6.1) library. The visualizations were performed using Plotly and Seaborn packages in a Python programming environment.

To handle missing data, all features and rows that contained more than 50% empty values were dropped. A threshold of 50% provided a balance between retaining as much data as possible. Additionally, rows that contained empty values in columns that were deemed not to be imputable were dropped. Lastly, rows that contained >50% empty values were also dropped, due to there not being enough data to impute them accurately. Any last empty values were imputed using a k-nearest neighbors (KNN) algorithm. The resulting dataset contained 53 features and 233 rows, consisting of 141 winning teams and 92 losing teams.

After the data preparation, an exploratory data analysis (EDA) was conducted to gain an initial understanding of the behavioral constructs and their association with team performance. The dataset was grouped by game outcome, that is, winning and losing teams, enabling comparative assessments across both groups. All 3 constructs, that is, collaboration, communication, and leadership, were analyzed by aggregating individual variables into relevant composite indices. For example, the scores of all behavioral indicators in the leadership construct were added to calculate the value of the leadership construct, and so on.

For each composite variable, statistical descriptors such as mean and SE around the mean were calculated and visually analyzed. In addition, statistical tests for significance were applied to compare the differences between winning and losing teams. The Student *t* test was applied using independent samples, where the assumptions of normality were satisfied, and Mann-Whitney U tests based on Shapiro-Wilk tests [29] were used where the assumption of normality was violated.

Subsequently, to examine the correlations between behavioral indicators, Pearson correlation analysis was applied and visualized using heatmaps. The correlation analysis quantified the linear associations between behavioral indicators, which were further quantified by fitting a regression model into the scatter plot of respective variable groups. The quantification of linear associations visualized the extent of the difference between behavioral indicators exhibited by the winning and losing teams. The EDA resulted in some interesting insights about underlying group differences between winning and losing groups in the escape room activities.

### ML-Based Modeling

In order to develop ML models for classifying between winning and losing teams, the preprocessed dataset was split into train and test sets in a proportion of 80% and 20%, respectively. Due to the unbalanced nature of the dataset, the split was stratified with respect to the target variable, which ensured an equal proportion of the target variable between the train and test sets.

All models were developed in the Scikit-Learn library (version 1.6.1) using the Python programming language (version 3.13.1) with necessary preprocessing steps, including feature scaling and feature selection. The purpose of feature selection was to enhance the performance of models and reduce potential overfitting. It was implemented using the SelectKBest method [30] with the chi-squared test as its scoring function to evaluate statistical relationships between each feature and the target variables (win or lose) and selecting only the most relevant features based on their chi-squared scores.

A selection of supervised classification models, namely: support vector classifier (SVC), KNN, Gaussian NB, RF, multilayer perceptron (MLP), gradient boosting classifier, logistic regression (LR), decision tree, adaptive boosting classifier, ridge classifier, and linear discriminant analysis was trained twice, once on unscaled dataset and once on a standardized dataset, and subsequently evaluated using a 5-fold cross-validation with *F*_1_-scoring. The hyperparameters were optimized using the GridSearch method, based on the highest 5-fold cross-validation with *F*_1_-scoring (≤3240 fits per model). The optimized hyperparameters were mostly related to the regularization complexity of the models. For SVC, these are C (regularization parameter) and kernel and gamma (kernel coefficient). For RF, these are number of estimators, maximum depth, and minimum samples needed for a split and a leaf. For MLP, the size of the hidden layers, the activation function, the solver, and the maximum allowed iterations. Lastly, LR was optimized on the C (regularization parameter), the penalty function, and the maximum iterations.

Lastly, the best-performing model was selected for interpretation and to identify the importance of each feature in shaping the final prediction. This was achieved by (1) extracting the model coefficients to analyze their weights associated with each feature and (2) the SHAP (or Shapley Additive Explanations) [31] values, which were determined using the SHAP library (version 0.48).

**Figure 5.**
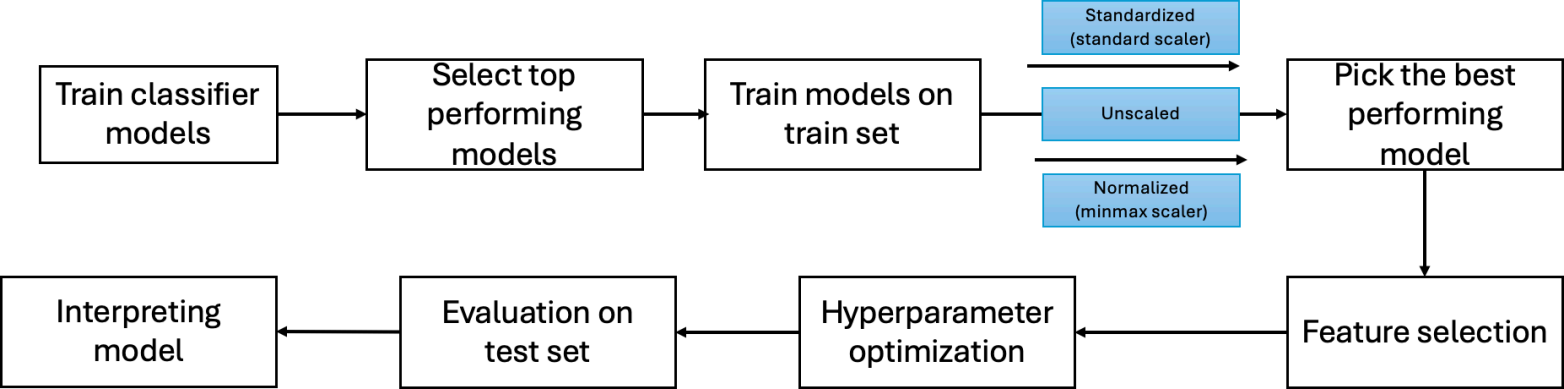
N - Model Development Workflow.

This study followed the TRIPOD (Transparent Reporting of a Multivariable Prediction Model for Individual Prognosis or Diagnosis) reporting guideline to ensure transparency and completeness in reporting the development and evaluation of the ML models [32]. A completed TRIPOD checklist has been included as a supplementary file (Checklist 1). The overall workflow used for developing, optimizing, and interpreting the ML models is illustrated in Figure 5.

### Ethical Considerations

This study involved secondary analysis of anonymized behavioral data collected independently by JGM Serious eXperiences as part of their standard team-training activities. The Campus Fryslân Ethics Committee confirmed that formal ethical approval was not required for this study. This determination is consistent with the committee’s review procedures, under which studies involving the secondary analysis of fully anonymized data with no identifiable information or participant interaction are classified as Tier 1 (no formal review required). The study was conducted in accordance with established ethical standards and institutional guidelines, including the World Medical Association Declaration of Helsinki and relevant data protection regulations such as the General Data Protection Regulation (GDPR). All data were handled in compliance with applicable privacy and confidentiality standards.

All participants who engage in JGM’s escape-room training activities are required to read and sign a written informed consent form before participation. Participants are informed about (1) the purpose of the activity, (2) the presence of trained observers, (3) the nature of the behavioral data collected, and (4) their right to withdraw at any time. The provided JGM consent form explicitly states that behavioral and observational data may be used anonymously for scientific research and that all identifying fields (name, age, and email) are removed before data storage and sharing. The consent form confirms that participants grant permission for the anonymized secondary use of their data for research purposes.

To protect participant confidentiality, JGM removes all personally identifying information before data are uploaded to their internal database, otherwise known as the team analyzer. The research team only received anonymized, team-level variables (eg, communication, collaboration, and leadership scores) with no access to names or any other identifying fields. All data was shared securely and stored on encrypted university servers. No reidentification of individuals was possible, as the research team had no access to raw video, audio, or personal data.

The research team did not compensate participants. Participation in JGM sessions occurred voluntarily as part of organizational training programs or recreational use, and JGM’s standard activity fees (if applicable) were unrelated to the research component. No images, videos, or audio recordings of participants are included in this paper or supplementary materials. Therefore, no consent for identifiable images was required. All analyses were conducted solely on anonymized numerical and categorical variables extracted from JGM’s internal behavioral coding system.

## Results

### Characteristics of Data

The final dataset consisted of 233 team-level observations, with an imbalanced distribution of outcomes: 141 teams completed the escape room game (ie, winning teams), whereas 92 teams did not achieve success (ie, losing teams). The team sizes varied between 2 and 8 members, where most of the teams consisted of 4 or 5 members. The smaller teams (2‐3 members) and the larger teams (6‐8 members) were relatively uncommon, which resulted in most of the teams in the dataset being midsized. The experience in escape rooms was also recorded on an individual level and then aggregated at the team level. A significant number of teams (n=126) reported no experience. In contrast, a smaller group (n=25) indicated moderate familiarity, and an even smaller number (n=9) reported a higher level of prior engagement.

The mean age of participants across all teams was 25.8 years. The majority of teams (n=107) had a mean age of 24.9 years, whereas only 6 teams had a mean age above 50. In terms of gender composition, 53 teams were composed entirely of male members, whereas 55 teams were entirely composed of female members. The remaining teams were composed of both genders, and the gender proportion varied significantly across groups.

In summary, the dataset captures a diverse sample of teams, mainly composed of younger participants, with limited prior escape room experience and mixed gender composition. These demographic and background characteristics provide context for interpreting teams’ performance in the escape room environment.

### About EDA

Overall, winning teams showed higher scores in environmental awareness, leadership, guidance, and extraversion, with CIs that were narrow and showed little overlap, indicating more reliable group differences. In contrast, constructs such as coordination, knowledge sharing, consensus, and method of communication showed overlapping CIs, suggesting that any mean differences between winners and losers are small and less precise.

For environmental awareness, the mean difference between winners and losers was 2.33 (95% CI 1.27 to 3.38). Although this suggests a noticeable gap, the Shapiro-Wilk test indicated nonnormality, and the Mann-Whitney *U* test showed no statistically significant difference (U=9595.00, *P*=.68). This means the construct does not reliably distinguish the 2 groups.

Knowledge sharing showed a smaller mean difference of 0.43 (95% CI 0.01 to 0.84). Despite marginal overlap in CIs, the Mann-Whitney *U* test revealed a statistically significant difference (U=13,687.50, *P*<.001), indicating that winners scored meaningfully higher than losers.

Leadership also showed a clear difference, with a mean gap of 1.21 (95% CI 0.46 to 1.95). Normality was violated, so a Mann-Whitney *U* test was used, and it confirmed a statistically significant difference (U=12,502.00, *P*<.001). Guidance showed a similar pattern, with a mean difference of 1.27 (95% CI 1.46 to 2.47) and strong statistical significance (U=14,577.00, *P*<.001).

Extraversion of communication had a mean difference of 0.77 (95% CI 0.28 to 1.25), also supported by a significant Mann-Whitney *U* result (U=12,347.00, *P*<.001). Other communication-related constructs showed weaker differences. Directness of communication had a mean difference of 0.55 (95% CI 0.10 to 0.99), but the Mann-Whitney *U* test showed no significant difference (U=10,842.50, *P*=.17). Consensus within the team slightly favored losers with a mean difference of −0.34 (95% CI −0.86 to 0.19), but again, results were not significant (U=8917.00, *P*=.16). Method of communication showed a small and nonsignificant mean difference of 0.35 (95% CI −0.03 to 0.74; U=10,842.50, *P*=.17).

Coordination showed virtually no difference between groups with a mean difference of −0.09 (95% CI −0.49 to 0.32). This was the only construct meeting normality assumptions for both groups, yet the Mann-Whitney *U* test still indicated no significant difference (U=9595.00, *P*=.68).

In summary, only 4 constructs: knowledge sharing, leadership, guidance, and extraversion, showed statistically significant differences between winners and losers. These findings, supported through CIs, normality testing, and appropriate nonparametric analyses, suggest that only a subset of behavioral indicators meaningfully distinguish successful teams.

**Figure 6.**
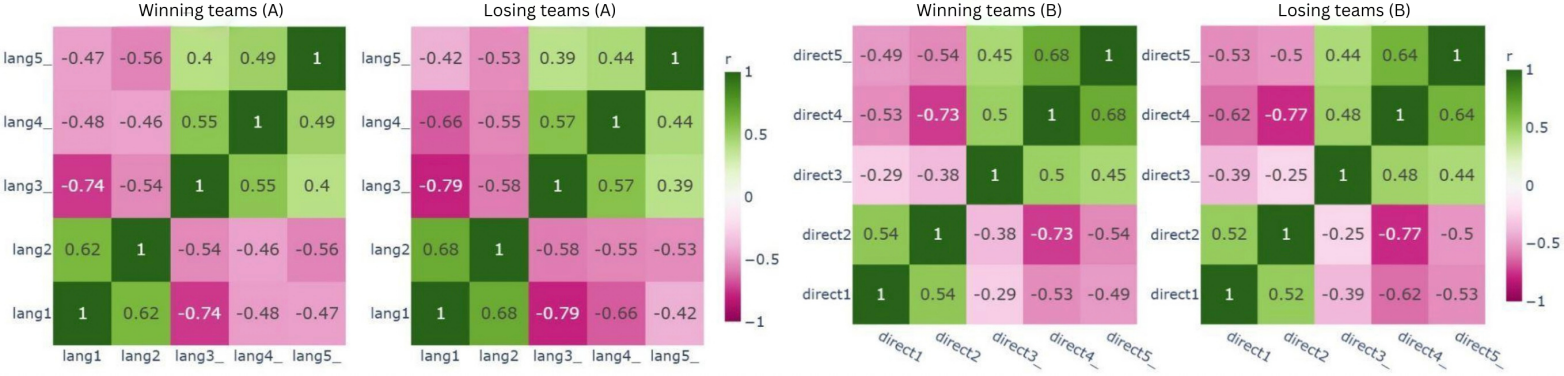
Pearson correlation for (A) communication and (B) directness of communication in winning and losing teams.

In addition, Pearson correlation analysis was used to analyze variable level relationships between the winning and losing groups. Figure 6 shows correlation maps for the communication construct. The winners displayed positive correlations between some individual variables (eg, lang1 and lang2, *r*=0.62) and negative correlations involving the lang3_ variable (eg, lang1 and lang3_, r=–0.74). In contrast, the losing teams mostly exhibited negative associations, suggesting a less differentiated structure.

Within the “directness of communication” construct, a negative correlation was found between direct4_ (use of only clean words) and direct2 (use of strong language, including swearing), with coefficients of r=–0.73 for winners and r=–0.77 for losing teams. As both groups show a similar pattern, this construct does not differentiate between winners and losers as shown in Figure 6B.

**Figure 7.**
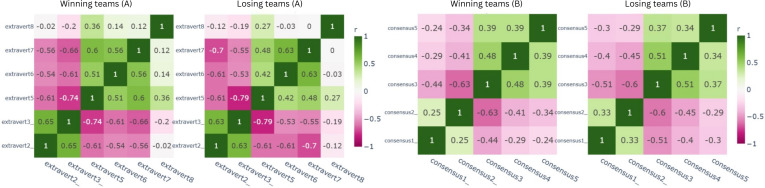
Pearson correlation for (A) extraversion of communication and (B) consensus-building in winning and losing teams.

This mirrored structure was also evident in other constructs. Figure 7A shows the extraversion of communication construct, extravert3 (lack of active communication) and extravert5 (continuous communication) were negatively correlated (r=–0.74 for winners; r=–0.79 for losing teams). Similarly, within the consensus construct in Figure 7B, consensus3 (waiting for team approval before acting) and consensus2 (seeking advice but acting independently) demonstrated a similarly negative correlation (r=–0.63 for winners; r=–0.60 for losing teams.

**Figure 8.**
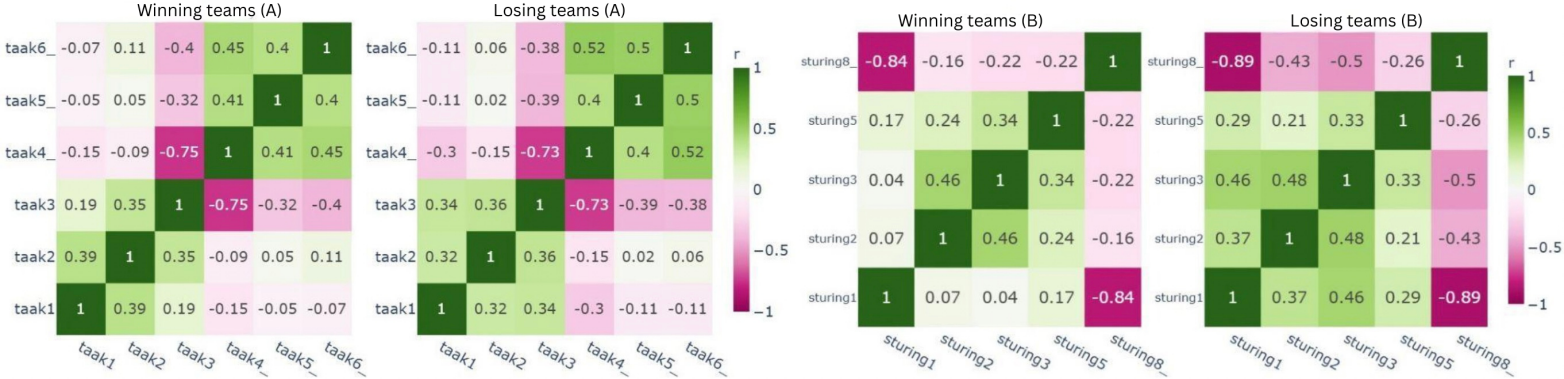
Pearson correlation for leadership and guidance in winning and losing teams.

Furthermore, Figure 8Adisplays variables in the leadership construct, showing taak4 (instructions are provided) and taak3 (group members give each other instructions) as inversely related, with correlation coefficients of r=–0.75 for winners and r=–0.73 for losing teams. Similarly, the guidance construct in Figure 8B also displayed redundant variables between sturing1 (there is group activity) and sturing8_ (the group has a wait-and-see attitude), with correlation coefficients of *r*=−0.84 for winners and *r*=−0.89 for losing teams.

**Figure 9.**
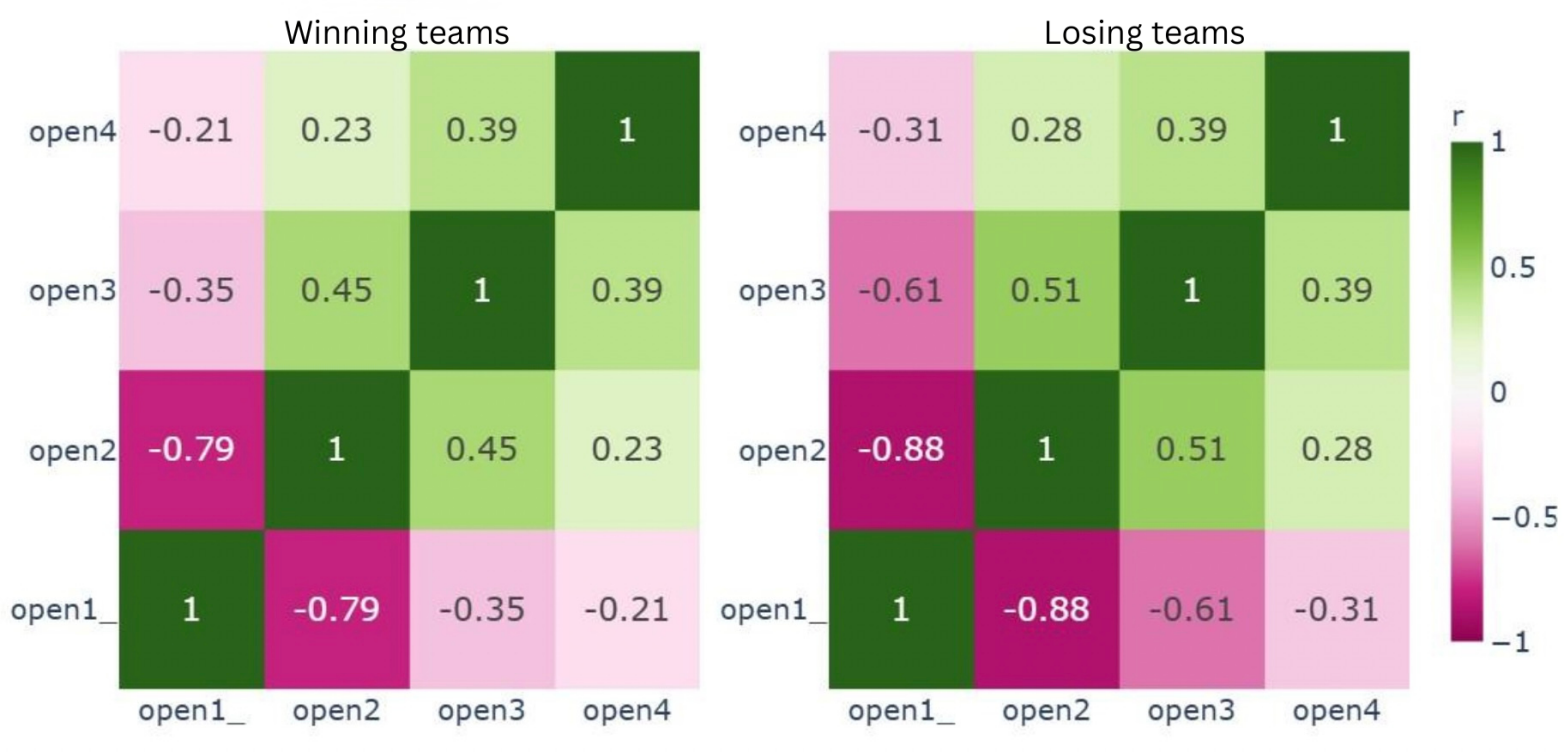
Pearson correlation for sharing knowledge in winning and losing teams.

The final construct showing overlap between variables was the sharing knowledge construct. For instance, open2 (knowledge is shared immediately with the group after learning about it) and open1_ (team members keep the knowledge they have gained to themselves) mirrored each other in winning teams, with correlation coefficients of *r*=−0.79 for winners and *r*=−0.88 for losing teams. These relationships are illustrated in Figure 9, which presents the correlation patterns within the sharing knowledge construct for winning and losing teams.

However, not all constructs exhibited opposing relationships. Constructs such as coordination within the team and environmental awareness showed generally inconsistent correlations among their variables, suggesting that these behavioral dimensions may capture more independent or multifaceted aspects of team dynamics. Unlike the previously discussed constructs, no clear inverse patterns emerged, indicating that the variables within these domains may reflect complementary rather than redundant behaviors.

Taken together, the exploratory analysis using descriptive statistics and Pearson correlation provided insights into group-level behavioral differences and variable relationships. Descriptive comparisons revealed that winners tended to score higher in constructs related to leadership, guidance, extraversion, and environmental awareness, whereas losing teams displayed less variation across several domains. Furthermore, the correlation analyses highlighted numerous instances of inverse relationships within constructs, such as communication, directness, extraversion, and leadership, suggesting the presence of opposing behavioral features.

### Performance of ML Models

Of the 12 models evaluated, 4 classifiers including LR, MLP, RF, and SVC, demonstrated consistently strong performance on *F*_1_-score across cross-validation. These models were subsequently selected for hyperparameter optimization and interpretation. The remaining models, including Gaussian NB, KNN, and others, showed substantially weaker performance and were therefore excluded from further analysis. Table 1 presents various evaluation metrics associated with 4 ML models developed to classify between winning and losing teams. The results were obtained by evaluating all 4 models on an unseen test set. In addition, 5-fold stratified cross-validation was performed using the *F*_1_-score as the accuracy metric (Table 1).

**Table 1.** ML^a^ model scores.

	Test-set scores				Cross-validation scores (*F*_1_-score)
Model	Accuracy (%)	Precision (%)	Recall (%)	*F*_1_-score (%)	Mean (SD, %)
Logistic regression	85	84	93	88	79 (0.037)
Support vector classifier	81	79	93	85	81 (0.044)
Multilayer perceptron	83	81	93	87	80 (0.039)
Random forest	83	81	93	87	80 (0.072)

a ML: machine learning.

Across the board, all 4 models yielded reasonable accuracies of over 80%. Interestingly, all models had an equal recall score of 93%, with slight differences in terms of precision score, where the LR model slightly outperformed the other models. The LR model also outperformed the other models in terms of accuracy and *F*_1_-score, which is a better representation of the model’s performance given a significant imbalance in the target variable. The SVC model accuracy was the lowest on the test set, but it outperformed all other models in terms of cross-validation score. On the other hand, the LR model scored the lowest score on the cross-validation metric (2 percentage points lower than SVC). However, the difference between the accuracies and cross-validation scores for all 4 models was not substantially different from each other (within 5% range).

**Figure 10.**
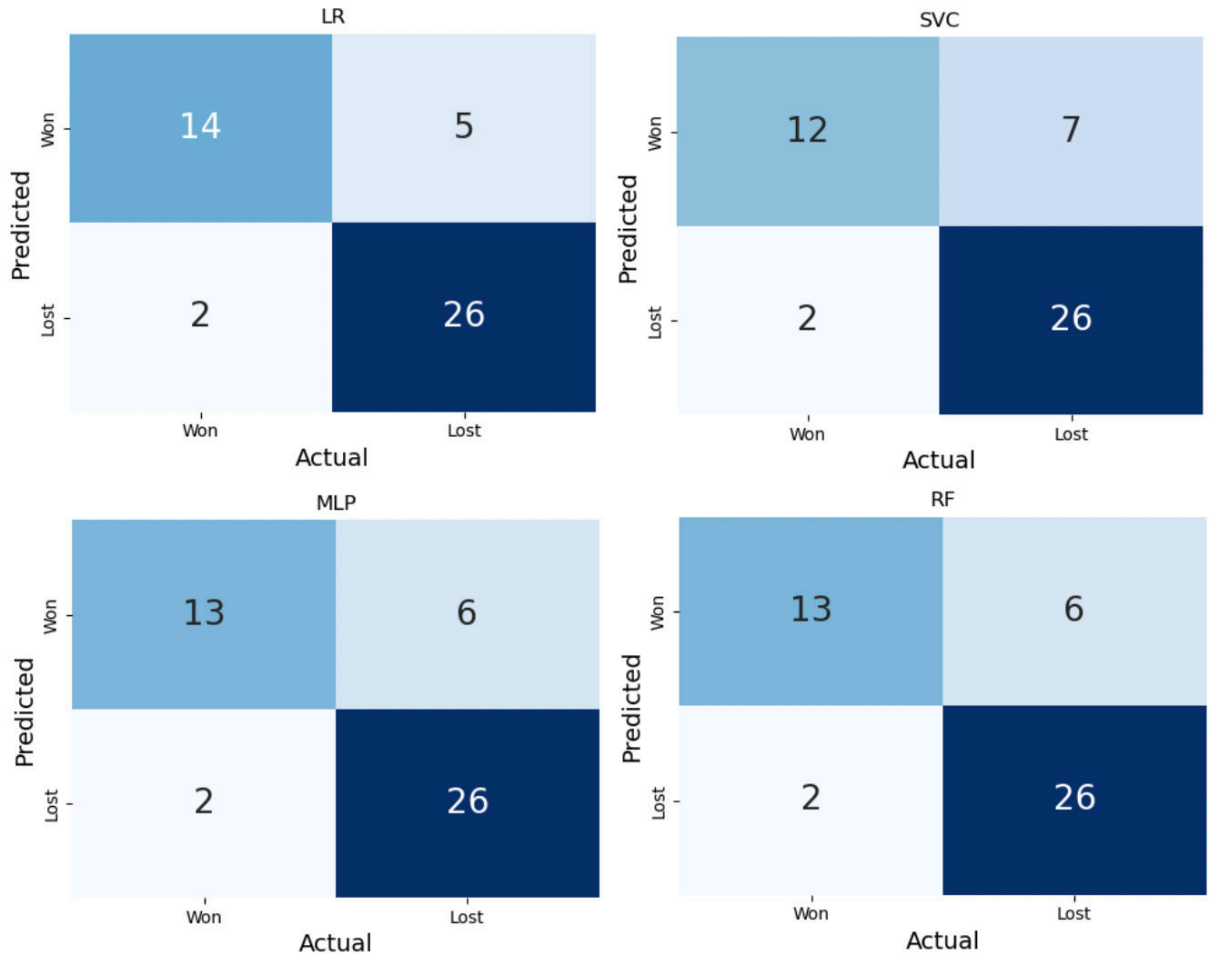
Confusion matrix for 4 models: LR, SVC, MLP, and RF. LR: logistic regression; MLP: multilayer perceptron; RF: random forest; SVC: support vector classifier.

Figure 10 presents the confusion matrix for the 4 ML models used to classify team outcomes as “won” or “lost” based on team behavioral indicators. The MLP and RF models both correctly identified 13 winning teams and 26 losing teams, with 6 false positives and 2 false negatives. The SVC model performed similarly, but with slightly more false positives (ie, 7) among winning classifications. In contrast, the LR model demonstrated the optimal balance, that is, correctly classifying 14 winning teams with only 5 false positives and 2 false negatives. Across all models, the number of false negatives stayed consistently low, but the number of false positives varied substantially. The LR model, in particular, achieved the optimal trade-off between sensitivity and specificity in predicting team success.

### Explainability of Model

**Figure 11.**
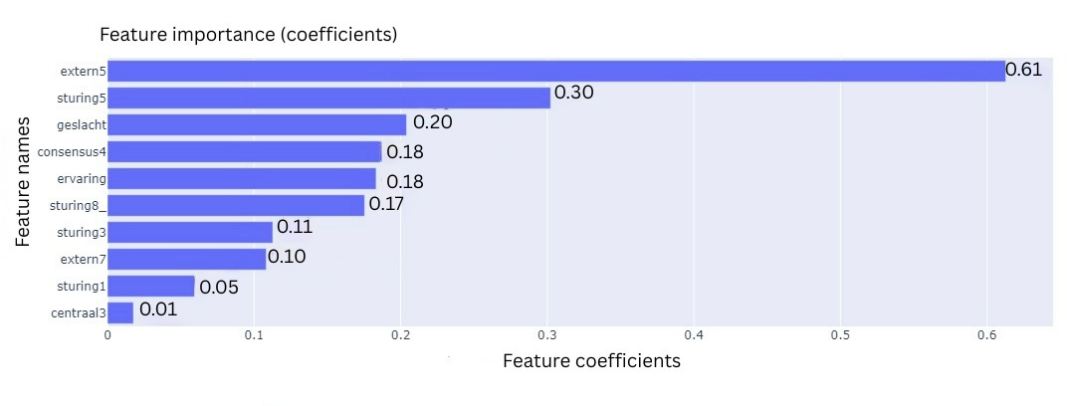
Feature importance of logistic regression

**Figure 12.**
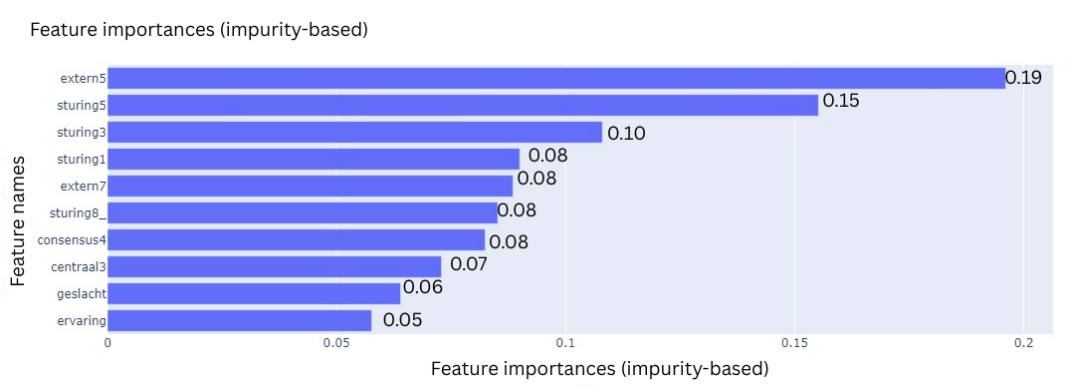
Feature importance of random forest.

**Figure 13.**
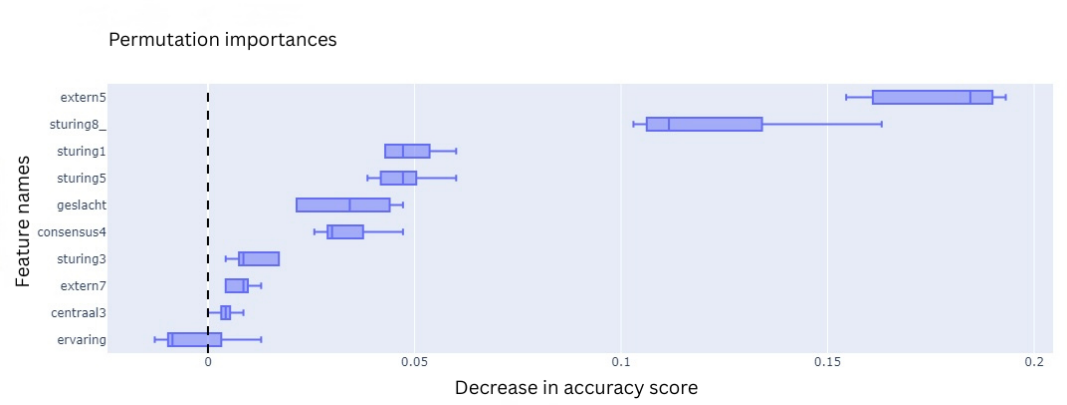
Permutation importance of the support vector classifier

**Figure 14.**
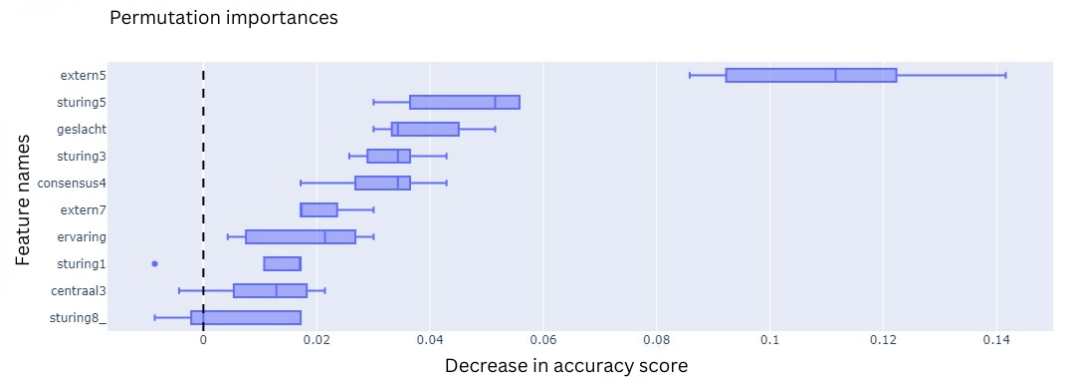
Permutation importance of the support vector classifier.

All 4 ML models were trained on 10 features that were selected during the feature selection process. To explain the contribution of each feature to the final classification outcome, the feature importances were extracted using various techniques based on model type. For the LR model, these are the coefficients, which are the internal weights of the model (Figure 11). For RF, its Gini impurities quantify how much a feature helped in decreasing uncertainty (Figure 12). The feature importances of MLP (Figure 13) and SVC (Figure 14) were quantified using permutation importances, a method that finds the most important features by shuffling feature inputs.

Across the models, extern5 (team members celebrating progression with each other) and sturing5 (someone taking the lead when the team is stuck) consistently ranked among the top features contributing to the prediction of team success. In the LR model, extern5 had the highest absolute coefficient, followed by sturing5, geslacht (gender distribution), and consensus4 (time to action after consensus). In the RF model, impurity-based feature importance ranked extern5, sturing5, sturing3 (guidance is provided through questioning and progress monitoring), and sturing1 (all team members are actively involved in tasks to find solutions as a group) as the top 4 features. In contrast, the MLP model showed higher permutation importance for extern5, sturing8_ (the team has a wait-and-see attitude), and sturing1, with sturing5 ranking fourth. For the SVC model, permutation importance highlighted extern5, sturing5, geslacht, and sturing3 as the most influential features. While the top-ranked features varied slightly across models, extern5 emerged as the most consistently important predictor.

**Figure 15.**
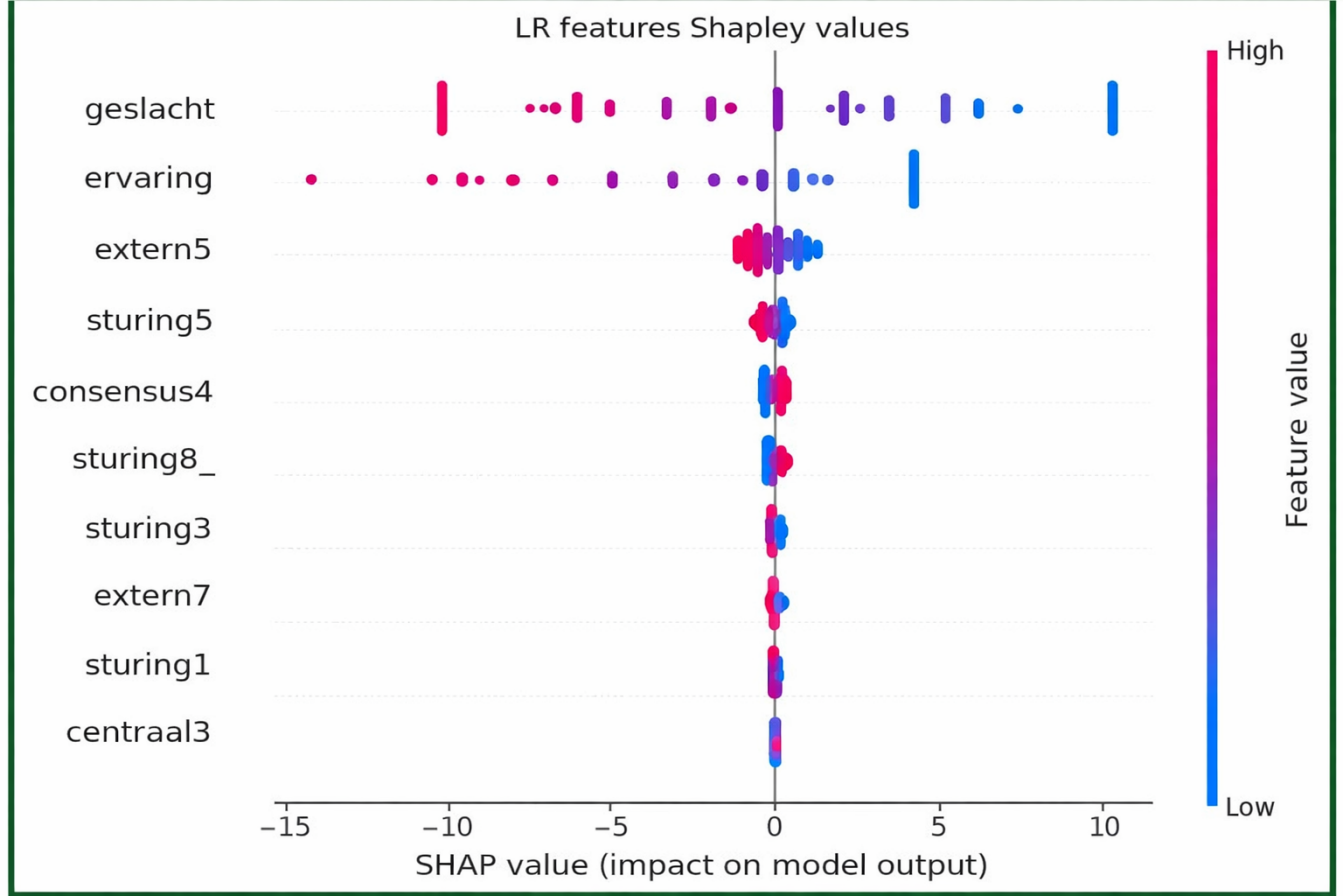
Shapley values of (best performing model) logistic regression.

In addition, SHAP values were computed for the best-performing model, that is, the LR model, to identify the features with the greatest impact on the model’s output (Figure 15). The most influential features included geslacht (gender composition) and ervaring (prior escape room experience), both of which showed the highest SHAP value ranges. These were followed by extern5 (celebrating progress) and sturing5 (taking the lead when the team is stuck), which had consistently positive SHAP contributions. Other features, such as consensus4 (waiting after consensus) and sturing8_ (passive behavior), exhibited SHAP values that were negatively associated with the model output, whereas sturing3, extern7, and sturing1 had comparatively lower but consistent influence on predictions. The SHAP summary plot thus reflects a combination of demographic and behavioral predictors, with varying degrees of contribution to the classification of team success.

## Discussion

### Principal Findings

This study set out to examine whether team success in SGs can be better understood through a multivariate combination of behavioral and demographic factors, rather than through isolated indicators. The findings show that team performance in escape room–based SGs is shaped by multivariate patterns of behavior. Successful teams were not distinguished by single actions alone, but by coordinated behavioral dynamics related to leadership, guidance, and expressive communication, alongside demographic characteristics such as team composition and experience. By applying exploratory analysis together with ML and explainable AI techniques, this study demonstrates that these factors jointly contribute to predicting successful team outcomes. Overall, the results confirm this study’s objective by showing that team success in SGs emerges from complex, multivariate behavioral patterns rather than from individual behavioral indicators considered in isolation.

### Interpretation of Behavioral Patterns in Team Performance

The exploratory analyses highlight clear behavioral differences between winning and losing teams in serious gaming environments. Winning teams tended to display stronger behavioral patterns related to leadership, guidance, environmental awareness, and expressive communication. These behaviors appeared more consistently and coherently among successful teams, whereas losing teams showed greater variability. In contrast, behaviors such as coordination and knowledge sharing were present in both groups but did not clearly differentiate successful from unsuccessful teams, suggesting that these behaviors alone are not decisive for performance in escape-room contexts.

Differences also emerged in how behaviors were combined within teams. Winning and losing teams often relied on similar behavioral elements, but they appeared to integrate them in distinct ways. For example, successful teams showed more balanced patterns of support, initiative, and shared progress, whereas losing teams tended to exhibit more uneven behavioral interactions. This indicates that team success may depend less on the presence of individual behaviors and more on how these behaviors interact during gameplay.

In particular, behaviors related to guidance and environmental awareness revealed contrasting interaction patterns between groups. While both winning and losing teams engaged in information seeking and guidance-related actions, losing teams appeared to rely more on reactive or delayed coordination, whereas winning teams demonstrated more adaptive behavioral combinations. These findings suggest that winning teams are not simply more active but are better at aligning leadership, guidance, and collective awareness in response to evolving game demands.

Overall, the exploratory findings indicate that team success in SGs is associated with multivariate behavioral patterns rather than isolated actions. Winning teams appear to combine leadership, guidance, and communicative behaviors in a more integrated manner, supporting the view that team performance emerges from multivariate behavioral dynamics rather than single behavioral indicators.

### Interpretation of ML and Explainable AI Findings

The ML analyses further support this multivariate interpretation of team performance. Across all 4 models, extern5 and sturing5 are overall the 2 most important features to determine whether a team will win or not. Extern5 refers to “team members celebrating progression with each other,” whereas sturing5 refers to “someone taking the lead when the team is stuck.” The significance of these 2 features displays the importance of good leadership and exuberance. The importance of the other features varied from 1 model to another and had a moderate impact on the final prediction. For the LR model, these were geslacht and consensus4, which reflect the “gender distribution of a team” and “the time it takes for a team to take action after consensus has been reached.” Sturing3 and sturing1 (which refer to “a team’s activity level” and “guidance provided”) were ranked third and fourth, respectively, with a moderate impact for the RF model. MLP was the only model for which sturing5 was not one of the two most important features, ranking only fourth after sturing8_ and sturing1, which also reflect a team’s activity level. Lastly, geslacht and sturing3 (which refer to “gender distribution of a team” and “guidance provided”) were the third and fourth most important features of the SVC model.

The SHAP explainable AI technique revealed that the most impactful features influencing team success were gender composition and escape room experience. Specifically, teams with a higher proportion of female members and greater experience were more likely to be classified as winners. The behavioral traits such as sharing positive emotions when progress was made (extern5) and guiding the team during uncertainty (sturing5) had a mild but positive influence on success. In contrast, passive or hesitant behaviors, such as waiting for others to act (consensus4 and sturing8_), tended to decrease the likelihood of winning. The model also found that early enthusiasm (extern7) and collaborative monitoring (sturing3) were weak but consistent predictors of better performance. These results suggest that successful teams managed uncertainty not simply by dividing tasks, but by enabling collaborative leadership, expressing collective motivation, and establishing situational awareness.

It is important to note that the key variables identified through quantification of feature importances of the ML models and those highlighted by the SHAP explainability technique were not always identical. This divergence is due to the fundamental differences in how these methods evaluate the relationship between predictors and the target outcome. The ML models rank features based on their predictive contribution to classification performance, which the algorithm’s internal mechanics influences [33] (eg, regularization in LR, impurity reduction in RF, or learned weights in MLP).

In contrast, SHAP values rank features in a model-agnostic way. They provide an estimate of the marginal contribution of each feature by simulating its effect across all possible combinations of feature inputs. SHAP estimates how much each feature contributes to the predictions by considering its effect across different combinations of inputs. In the results, SHAP highlighted demographic factors such as gender distribution and experience, whereas the ML models gave more weight to behavioral indicators such as extern5 and sturing5. Together, these methods offer complementary perspectives, showing that both demographic and behavioral factors played a role in shaping team outcomes. Recognizing these methodological differences is critical for interpreting the results holistically, as each method offers complementary insights into what drives team success. In sum, predictive models compute outcomes, whereas SHAP explains the underlying contribution of features to those outcomes [33].

### Comparison With Existing Literature

Prior work [34] emphasized the importance of team familiarity and communication across subgroup faultlines in shaping performance. Using survival models, they found that team familiarity could exert a negative influence on success, particularly when communication across subgroups was limited [34]. While this study did not explicitly model faultlines or social ties, it identifies behavioral and demographic features that contribute to team success, potentially through similar mechanisms of coordination, engagement, and leadership. For instance, sturing5 (taking initiative when the team is stuck) and sturing3 (guiding others and monitoring progress) reflect adaptive leadership and teamwork, which help teams stay on track, especially in unfamiliar or fast-paced situations [34]. Furthermore, a study [27] conducted one of the few observational studies into collaboration within escape rooms, emphasizing the central role of verbal communication, implicit leadership emergence, and social awareness. Their qualitative insights on players communicating loudly across the room, navigating spatial awareness through verbal cues, and different leadership styles [27] echo the behavioral constructs we identified as significant. For example, their observations that experienced players often assume leadership roles [27] align with the predictive power of ervaring (experience) and sturing5 (leading when stuck) in our models. Their finding that teams converge when stuck [27] also aligns with the identification of sturing1 (early active problem-solving) and sturing3 (task-related guidance) as positive predictors.

Beyond the context of SGs, our findings are aligned with a broader trend in applied ML, where complex, multifactor systems are modeled using data-driven and often hybrid approaches. Recent studies in smart city energy forecasting, groundwater management, CO₂ emissions prediction for electric vehicles, and rainfall classification have combined traditional statistical models or neural networks with metaheuristic optimization and feature-selection techniques to handle high-dimensional, noisy, and context-dependent data while improving predictive accuracy and efficiency [35-39]. In a similar sense, our study uses EDA, supervised ML, and explainable AI to identify a set of behavioral and demographic predictors that capture the significant variables of team performance in escape rooms. Rather than focusing on metaheuristic optimization [35,37], we emphasize data preprocessing, feature selection, and interpretability as practical strategies for organizations that work with limited or expert-coded data. This positions our work within a growing body of research showing that ML techniques can reveal meaningful patterns in complex systems, whether environmental or behavioral, when combined with domain knowledge and transparent model evaluation. These methodological parallels across domains illustrate a broader trend in ML toward transparent, data-driven analysis, which aligns with the approach taken in this study.

This study makes several contributions to research on team performance in SGs. Methodologically, it advances the field by combining EDA, supervised ML, and explainable AI to examine team success as a multivariate behavioral phenomenon rather than as the outcome of isolated factors. This approach differs from much of the existing serious-games literature [10-13,15,17,21], which has primarily relied on univariate statistical comparisons or qualitative observations [27] and has rarely integrated interpretable ML to explain why certain teams succeed. By identifying multivariate influential behavioral and demographic predictors across multiple models, the current study contributes empirical evidence on which observable team behaviors meaningfully distinguish successful from unsuccessful teams in escape-room settings, which can be translated in terms of improved productivity in business and organization settings.

### Implications for Understanding Team Performance in SGs

These findings have several implications for the design, evaluation, and application of SGs as tools for team training and assessment. First, identifying which behavioral patterns meaningfully distinguish successful from unsuccessful teams can support the refinement of observer-coded behavioral frameworks. Rather than relying on a large number of overlapping indicators, SG designers and expert observers can focus on a smaller set of behaviors that capture leadership, guidance, and collective engagement more effectively. This may improve the efficiency of behavioral assessment in game-based training environments.

Second, the results can inform the selection of performance indicators used during and after gameplay. By highlighting behavioral combinations associated with success, SGs can move beyond simple outcome measures, such as winning or losing, toward richer evaluations of how teams function under pressure. This opens opportunities for developing feedback tools that emphasize team strengths and areas for improvement, either through debriefings or automated postgame reports. Such debriefings can support reflection, learning, and skill development by making abstract team dynamics more concrete and observable.

Finally, this study demonstrates the practical value of interpretable ML for translating complex behavioral data into actionable insights. By combining predictive models with explainable techniques, it becomes possible to generate transparent and understandable explanations of team performance rather than opaque scores or rankings. This is particularly important in educational and organizational settings, where trust, accountability, and learning are central concerns [40,41]. Interpretable ML thus offers a promising pathway for integrating data-driven analysis into SGs in a way that supports understanding, feedback, and responsible decision-making.

### Limitations of Research

While the findings in this study provide some new insights into team performance in escape room–based serious gaming, several limitations need to be acknowledged.

First, the limited sample size could restrict the generalizability of our results. Additionally, with a significant class imbalance toward losing teams (141 losing teams and 92 winning teams), this could have introduced bias in the model for that specific group. This imbalance could bias model training because standard ML algorithms often assume that classes are equally represented. Prior research shows that when 1 class is larger, models tend to favor the majority class, leading to poorer detection of the minority class or even treating minority cases as noise [42,43]. Class imbalance is a well-known challenge across many applied domains, such as fraud detection, medical diagnosis, and intrusion detection, and can distort decision boundaries, reduce sensitivity to the minority group, and limit overall model fairness [42,43]. Although several methods have been proposed to manage imbalance, there is no universal solution suitable for all datasets [43]. In this study, the unequal distribution between winning and losing teams may therefore influence model performance and should be considered when interpreting the results.

Second, the limitation concerns the dataset size. Although machine-learning studies in fields such as image recognition often rely on datasets with tens of thousands of samples, behavioral research involving team performance rarely allows for such scale due to practical and resource constraints. Our dataset of 233 teams reflects these real-world limitations. As a result, model generalizability is reduced, and findings should be interpreted as proof of concept rather than fully generalizable predictions. Future research with larger, multi-institutional samples would be valuable for validating and extending these insights.

Third, the dataset is geographically and culturally limited to teams recruited in a specific demographic region. The cultural norms around communication, leadership, and emotional expression may influence gameplay behavior in ways that are not generalizable to teams in other countries or contexts. As a result, the applicability of the findings to international, intercultural, or remote teams may be constrained to 1 specific region only.

Additionally, this study focuses exclusively on a single game genre (escape rooms) within 1 specific serious gaming environment. While this provides insight into a certain domain of games, it might have limited transferability to other SGs involving different structures and dynamics, for example, simulations, role-playing, or strategy-based games, where behavioral markers and success criteria may differ substantially.

Finally, it is important to note that the conclusions drawn from this study are based on a cross-sectional retrospective design and therefore reflect associative and predictive relationships rather than causal effects. While the ML models identify behavioral and demographic patterns that are strongly associated with team success, they do not imply that modifying individual behaviors will necessarily lead to improved outcomes. Instead, the findings should be interpreted as evidence of multivariate patterns that characterize successful teams within the observed serious gaming context. Within these methodological boundaries, this study provides a robust, data-driven foundation for understanding team dynamics and for informing the design of feedback, training, and adaptive systems in SGs.

### Ethical and Interpretability Implications

This study relied on the secondary use of fully anonymized behavioral data collected by JGM Serious eXperiences as part of routine training activities. All participants provided written informed consent, all identifying information was removed before data storage and sharing, and the research team received only deidentified, team-level variables. .

Predictive analytics can infer sensitive traits or behavioral tendencies from seemingly harmless data, a concern described as “predictive privacy” [44]. This occurs when models generate inferences about individuals or groups without their awareness, potentially leading to differential treatment or unintended consequences. While our study does not involve such high-stakes decisions, the concept remains relevant for future applications of ML-based team assessment in organizational settings.

Another key issue concerns fairness and bias. Predictive systems can unintentionally reproduce existing social inequalities if model outputs correlate with attributes such as gender, experience, or group composition [44,45]. Demographic variables such as gender composition and experience were influential predictors in this study, reflecting patterns present in the dataset rather than normative judgments. SHAP values were used in this study to provide transparent explanations of model outputs, improving clarity around why certain predictions were made. Prior research shows that explainable AI can increase trust and informed decision-making in human-AI systems [40], whereas poorly explained feedback may negatively affect user engagement and performance [41]. As ML-based evaluation becomes more common, clear ethical safeguards and transparent design are needed to ensure that behavioral insights support learning rather than unfairly labeling participants.

### Conclusion

This study demonstrates that team success in serious gaming environments is best understood as a multivariate phenomenon emerging from the interaction between behavioral dynamics and team composition, rather than from isolated actions or traits. By integrating exploratory analysis with ML and explainable AI, the findings show that leadership, guidance, collective engagement, and contextual team characteristics jointly shape performance outcomes under time pressure. More broadly, this study highlights the value of treating SGs not only as training tools but also as structured environments for observing and analyzing complex team behavior using transparent, data-driven methods.

Beyond prediction, the results have broader implications for how SGs and AI-supported assessment systems are designed and deployed. Explainable AI techniques (eg, SHAP) enable behavioral data to be translated into understandable and actionable insights, supporting feedback, reflection, and learning rather than opaque scoring. Consistent with prior work showing that transparency and explainability influence how humans engage with AI systems [40,41], this study suggests that AI-supported SGs can enhance team development when human behavioral insight and explainable analytics are combined responsibly. In this way, the work contributes to a broader understanding of how human-AI systems can support effective teamwork, not by replacing human judgment, but by augmenting it with interpretable, evidence-based insight.

## Supplementary material

10.2196/83478 Checklist 1 TRIPOD checklist.

## Abbreviations

AI: artificial intelligence
ANTS: Anesthetists’ Non-Technical Skills
EDA: exploratory data analysis
KNN: k-nearest neighbor
LR: logistic regression
ML: machine learning
MLP: multilayer perceptron
NB: Naive Bayes
RF: random forest
SG: serious game
SHAP: Shapley Additive Explanations
SVC: support vector classifier
TRIPOD: Transparent Reporting of a Multivariable Prediction Model for Individual Prognosis or Diagnosis
